# Experimental and Numerical Studies on Fiber Deformation and Formability in Thermoforming Process Using a Fast-Cure Carbon Prepreg: Effect of Stacking Sequence and Mold Geometry

**DOI:** 10.3390/ma11050857

**Published:** 2018-05-21

**Authors:** Daeryeong Bae, Shino Kim, Wonoh Lee, Jin Woo Yi, Moon Kwang Um, Dong Gi Seong

**Affiliations:** 1 Advanced Materials Engineering, University of Science and Technology (UST), 217 Gajeong-ro, Yuseong-gu, Daejeon 34113, Korea; drbaeuk@kims.re.kr; 2 Korea Institute of Materials Science (KIMS), 797 Changwon-daero, Changwon, Gyungnam 51508, Korea; yjw0628@kims.re.kr (J.W.Y.); umk1693@kims.re.kr (M.K.U.); 3 Korea Aerospace Industries (KAI), 78 Gongdanro 1-ro, Sanam-myeon, Sacheon, Gyungnam 52529, Korea; shino.kim@koreaaero.com; 4 School of Mechanical Engineering, Chonnam National University, 77 Yongbong-ro, Buk-gu, Gwangju 61186, Korea; wonohlee@chonnam.ac.kr; 5 Department of Polymer Science and Engineering, Pusan National University, 2 Busandaehak-ro 63beon-gil, Geumjeong-gu, Busan 46241, Korea

**Keywords:** thermoforming, prepreg, carbon fiber, fast-cure epoxy resin

## Abstract

A fast-cure carbon fiber/epoxy prepreg was thermoformed against a replicated automotive roof panel mold (square-cup) to investigate the effect of the stacking sequence of prepreg layers with unidirectional and plane woven fabrics and mold geometry with different drawing angles and depths on the fiber deformation and formability of the prepreg. The optimum forming condition was determined via analysis of the material properties of epoxy resin. The non-linear mechanical properties of prepreg at the deformation modes of inter- and intra-ply shear, tensile and bending were measured to be used as input data for the commercial virtual forming simulation software. The prepreg with a stacking sequence containing the plain-woven carbon prepreg on the outer layer of the laminate was successfully thermoformed against a mold with a depth of 20 mm and a tilting angle of 110°. Experimental results for the shear deformations at each corner of the thermoformed square-cup product were compared with the simulation and a similarity in the overall tendency of the shear angle in the path at each corner was observed. The results are expected to contribute to the optimization of parameters on materials, mold design and processing in the thermoforming mass-production process for manufacturing high quality automotive parts with a square-cup geometry.

## 1. Introduction

As fuel economy regulations in the automotive industry become tighter [1], carbon fiber reinforced plastic (CFRP) parts and their processing are more attractive because the parts have lighter weight with better mechanical properties than steel parts while still maintain the durability and safety as the vehicle parts. To commercialize CFRP parts, a cost-effective mass production line must be developed. The ideal cycle time for a production line would be between 3 and 7 min per part. One of the forming technique that meets the above criteria is prepreg compression molding (PCM), which is also known as a thermoforming (or hot stamping) process [2]. Several studies have investigated the formability of a carbon fiber/thermoplastic prepreg on a hemisphere. The behavior of the fiber reorientations due to the fiber deformation from the two-dimensional prepreg to the three-dimensional mold shape during the thermoforming process [3,4] was studied to observe mechanical properties of the final product. The studies concluded that the fiber deformation behavior in the formed product was determined by the fiber orientation of the prepregs and the mold profile. Thermoforming processes with asymmetric L-shape part and square box shape were also studied to compare the computed shear angle values with the experimental results [5,6]. Analysis of thermomechanical behaviors such as in-plane shear and bending stiffness for thermoplastic based composites was conducted to understand the formation of wrinkles in the final thermoformed product [7,8,9]. Non-linear thermomechanical properties of prepreg at various deformation modes including frictional stress as intra-ply shear were also investigated [10,11,12]. Some benchmark studies were also carried for standardization of the test methods for measuring thermomechanical properties of prepreg in order to improve the accuracy of thermoforming process simulation [13,14].

However, only a few studies on fast thermoforming process by using fast cure thermoset resin were identified and there was a lack of research on the effect of the formability of the carbon fiber/epoxy prepregs on variations in the mold geometry and stacking sequence of laminate [15]. In automotive industries, the geometries of the CFRP-based vehicle parts, such as the roof, hood and trunk, are based on a flat quadrangular (square, rectangular or trapezoid) plane with extreme curvatures on the edge. The curvatures are known to be the most challenging section of the part to form and it is likely to create distortions during thermoforming process. Consequently, experimental studies aimed at high-speed compression molding of automotive parts are required to investigate the formability of the various stacking sequences of prepreg with different mold geometric shapes using a fast-cure epoxy resin. A simulation of forming process is also necessary to predict the formability of the part, which leads to the optimization of the process parameters for defect-free thermoformed product in advance.

In this work, highly accurate methods and testing devices to measure the non-linear mechanical behaviors of fast-cured carbon or glass fiber/epoxy prepregs with different woven patterns were prepared, which were used to measure the tensile, intra- and inter-ply shear properties of prepregs. The optimal processing temperature and curing time for the prepreg were determined by measuring the rheological and thermal properties of the resin [16]. Two different mold types with square-cup geometries were prepared and thermoforming tests were conducted to evaluate the fiber deformations and formability of the prepreg based on the draft angle and depth of the mold. After the forming stage, the in-plane shear deformation in the final parts was quantified. The commercial virtual forming simulation software (PAM-FORM) with input data obtained from the mechanical property tests was used to simulate the effect of the square-cup geometry and different stacking sequences of the prepreg layers on the shape and property of the final product, which was also compared to the experimental results. It is expected that our cumulative experimental and simulation results can provide a design guide for the fast-cure type prepreg thermoforming process. 

## 2. Experimental

### 2.1. Materials and Sample Preparation

Three types of prepreg were provided by Hankuk Carbon Co., Ltd., Miryang, Korea. The specifications of the prepregs are shown in Table 1. The prepregs consisting of plain woven (PW), unidirectional (UD) carbon or glass fibers were specifically formulated with a fast-cure, BPA-type thermosetting epoxy resin. 

In the forming stage, ten-layered laminates with symmetrical stacking sequences with respect to the mid-plane were prepared. Each layer was cut into a 300 mm × 300 mm square at various fiber angles (0/90°, −45°/+45°). Fiber angles were measured based on the rolling direction of the prepreg as the reference point. Table 2 illustrates two different laminate stacking sequences for verifying the formability of the square cup. Ten repeated measurements for the thickness of each prepreg were conducted by using an optical microscope (ECLIPSE LV150N, Nikon, Tokyo, Japan).

### 2.2. Measurement of the Non-Linear Mechanical Behaviors of the Prepreg at Elevated Temperatures

The optimum temperature for prepreg forming process is the temperature at which the resin has the lowest viscosity. Mechanical behavior of prepregs during the tensile [13], in-plane shear (intraply) [14] and frictional (interply) tests [10] may be affected by the flowability of the resin at elevated temperatures. Therefore, the developed methods and apparatus were introduced to characterize the different types of prepreg.

Although standardized methods to measure the tensile properties of the prepreg at elevated temperatures are unavailable, the specimen with an appropriate size were prepared for the tensile test. The overall length of the specimen and the gauge length were fixed at 240 mm and 100 mm, respectively. All the tests were performed at a cross-head speed of 10 mm/min and a chamber temperature of 100 °C. To minimize the temperature variation of the specimen, chamber was pre-heated for 5 min. After fixing the specimen in the grips, temperature was stabilized until required temperature was set. For the UD 90° carbon specimen, the tests were conducted under a low load using a dynamic mechanical analysis (DMA). The temperature stabilization procedure for the specimen was conducted for 5 min before starting the experiment. To overcome slippage at the end of the specimen during the tensile test, glass reinforced epoxy tabs with sandpaper were attached to both sides of the fabric, which can increase the frictional force between the tab and the fabric.

Several layers of prepreg require that the prepreg deforms against a particular design geometry without any wrinkles. This can be achieved via intraply shear within the prepreg ply and interply shear in-between the prepreg plies [13]. Interply shear deformation due to friction can be described by measuring the coefficient of friction for the prepreg-prepreg and prepreg-mold surfaces. The forming resistance created by a high coefficient of friction can cause wrinkles and built-in residual stresses within a stack of prepreg [14]. A suitable model to describe the interply shear deformation via friction at an elevated temperature is the hydrodynamic model [15]. The coefficient of friction is required to measure the friction between the two plies separated by a thin layer of low viscosity resin (fluid). Frictional force between the specimen and the tool surface was measured by using the as-made measuring apparatus with load cell, a pneumatic cylinder and step motor, which has been described in our previous research [17]. Preheating procedures for specimen was required to provide a constant viscosity of resin inside the prepreg. The mold temperature was set at 100 °C for a constant epoxy resin viscosity. The specimens were prepared with a length of 180 mm and a width of 85 mm and attached to both sides of the steel frame. To define the fiber orientation angle of the specimen, the horizontal movement direction of the test frame was designated as a reference line.

**Figure 1:**
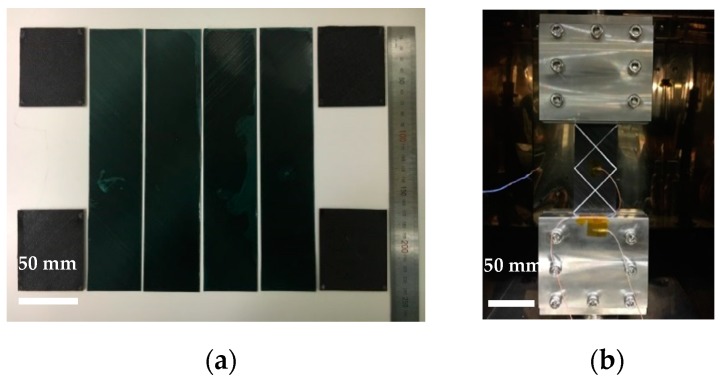
(**a**) Sample preparation and (**b**) test apparatus for the bias extension test.

A bias extension test [7,8] was conducted to identify the intra-ply shear properties of three different prepregs under the forming condition of 10 mm/min at 100 °C. Cross-piled fabrics +45° with −45° of UD, PW carbon and PW glass (50 mm × 240 mm) were used to keep the specimen in a symmetrical shape during the tensile test using the Shimadzu test machine and heating chamber (Figure 1a,b). Procedures for preheating and temperature stabilization inside the chamber were identical with the high temperature tensile tests. 

After obtaining shear angle from bias extension test, force-shear angle curve can be plotted. In order to represent the relationship between the normalized shear force and shear angle, the torque per original unit area can be calculated by using Equation (1) and an iterative process with Equation (2) [14]. The shear modulus can be calculated by measuring the slope of the shear stress-strain curve.

(1) $$C_{s}\left(\gamma \right)=F_{sh}\left(\gamma \right)\cdot \cos\gamma ,$$

(2) $$C_{s}\left(\gamma \right)=\frac{1}{\left(2H-3W\right)}+\left(\left(\frac{H}{W}-1\right)F\left(\cos\frac{\gamma }{2}-\sin\frac{\gamma }{2}\right)-WC_{s}\left(\frac{\gamma }{2}\right)\right),$$

where *C_S_*(γ) is the torque per original unit area that is needed to deform the fabric in shear, *F_sh_* is the normalized shear force and F is the power made through the clamping force.

The bending stiffness of the carbon fiber reinforced thin prepreg is generally very low compared to tensile strength due to the sliding between fibers and between yarns [18]. Therefore, the bending resistance of prepreg is considered as a less important factor during the forming process. Membrane element approaches were also used in macroscopic forming simulation to neglect the bending stiffness [19]. However, Liang et al. suggested that bending stiffness was a significant factor to describe wrinkle formation during the forming simulation. The author also proved that the number and size of the wrinkles obtained in the simulation results were caused by different level of the bending stiffness [20]. In this article, the formability of the prepreg with different tilting angles and material lay-ups has been emphasized to analyze especially corner and edge sections of thermoformed product rather than the formation of wrinkles on the main outside section of the part. Since the magnitude of bending stiffness is required as input data for simulation, results of calculated bending stiffness by a vertical cantilever test with a linear actuator and load cell at 90 °C from Alshahrani et al. for woven carbon/epoxy prepreg were used in the forming simulation [21]. Rheological behavior of epoxy resin and test parameters such as temperature and forming speed used in Alshahrani’s work were very similar to this work, which enabled us to use their test results on bending stiffness.

### 2.3. Mold Geometry

Two types of molds with a rectangular shape were prepared with tilting angles and drawing depths to investigate the effect of the geometric shape of the mold and punch on the prepreg formability. It was thought that the formability and demoldability of the prepreg would be affected by the drawing depth and angle. The drawing depth and tilting angle for the square-based Type 1 mold were 20 mm and 110°, respectively. The maximum width of the Type 1 mold was 100 mm and the minimum width at the bottom of the mold was 85.44 mm. A 100 mm × 100 mm square-based Type 2 mold had a 40 mm drawing depth with a 90° tilting angle. The corner radii of both the mold and square punch were 4.7 mm and 2.7 mm, respectively. The clearance between the punch and the mold was 2 mm, which was the target thickness for the thermoformed square-cup.

### 2.4. Thermoforming Apparatus and Procedure

**Figure 2:**
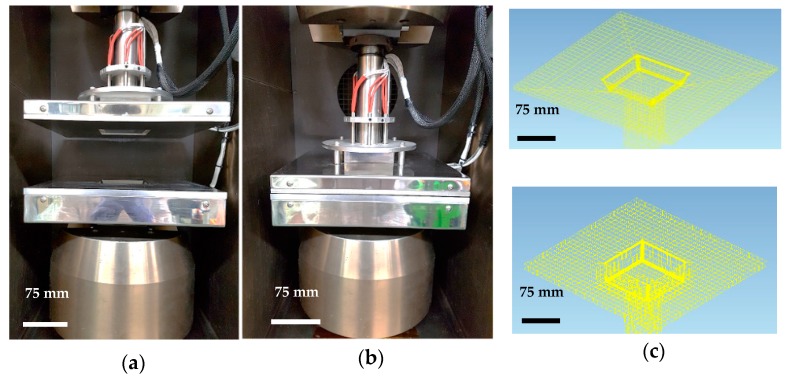
Thermoforming experimental apparatus for (**a**) open, (**b**) closed square-cup mold, (**c**) Type 1 with a 20 mm mold thickness and 110° draft angle (**top**) Type 2 with a 40 mm mold thickness and 90° draft angle (**bottom**).

The overall experimental apparatus for the thermoforming of the mold is illustrated in Figure 2. A 250 kN universal testing machine with an environmental convection chamber made by Instron 5985 (Instron, Norwood, MA, USA) was used to provide a constant punch forming speed under controlled environmental conditions. Temperatures of the electrically heated blank holder, mold, punch and chamber were individually controlled using cartridge heaters, which were kept at 100 °C. It was thought that thermoforming process with an isothermal condition would be advantageous to high-speed manufacturing process for automotive parts. If thermoforming process with multi-stage temperature conditions were used in the automotive industries, time required to raise and lower the mold temperature would be much longer than isothermal conditions. The forming speed was set to 10 mm/min and a constant weight of 10 kg (blank holder) was used throughout the experiment to minimize the wrinkles on the surface of the prepreg. The lubricant demolding agent for the thermosetting polymer was applied to the mold, punch and blank holder were dried at 60 °C for 30 min.

The procedure of prepreg square cup isothermal forming test is as follows. Ten-layered laminates of prepreg with the previously described stacking sequence were placed on the lubricant-coated base mold. The blank holder and punch were positioned at 2 mm from the top of the prepreg layer surface. The displacement of the punch was set to zero and the temperature of the mold, blank holder, punch and chamber was raised to 100 °C. As soon as the temperature reached 100 °C, the forming started with a 10 mm/min punching speed until the desired depth of the mold was reached. The final stage was to cure the prepreg at 120 °C for 10 min and cool to room temperature for demolding.

### 2.5. Forming Simulation

In order to fabricate a complex shape of final product without defects, the process simulation should be conducted. One of the challenges in thermoforming is the fiber deformations during the process, which may affect the mechanical performance of the final composite part. The commercial finite element (FE) code, PAM-FORM, provides a validated methodology and constitutive model for describing unidirectional and fabric sheets pre-impregnated by thermoset or thermoplastic resin. The composite material model in the PAM-FORM was used to describe the realistic behavior of prepreg including the fabric and unidirectional fiber structure [22,23,24]. Pre and post locking shear modulus can be defined by locking angle between warp and weft direction of fiber due to the shear force. Viscous friction law may be considered with temperature variations. Maxwell model in parallel with two linear elastic fiber phases is used for the mathematical material model. Heat transfer within the prepreg ply and ply-tool and heat conduction through shell elements are also modeled [25]. The constitutive law for plane stress of textile and resin materials can be defined as Equation (3).

(3) $$\left(\begin{array}{c} \sigma_{11}\\ \sigma_{22}\\ \sigma_{12} \end{array}\right)=\left(\begin{array}{cc} E_{11} & \varepsilon_{11}\\ E_{22} & \varepsilon_{22}\\ G_{12} & \varepsilon_{12} \end{array}\right)+\left[\begin{array}{ccc} 4\eta_{L} & 2\eta_{T} & 0\\ 2\eta_{T} & 4\eta_{L} & 0\\ 0 & 0 & 2\eta_{L} \end{array}\right]\left(\begin{array}{c} {\dot{\varepsilon }}_{11}\\ {\dot{\varepsilon }}_{22}\\ {\dot{\varepsilon }}_{12} \end{array}\right),$$

The first term of this equation is related to the elastic contribution of fiber and the second term includes the longitudinal (*η_L_*) and transverse (*η_T_*) terms of viscosity in either isothermal or temperature-dependent forming conditions.

## 3. Results and Discussion

### 3.1. Material Properties of the Resin

**Figure 3:**
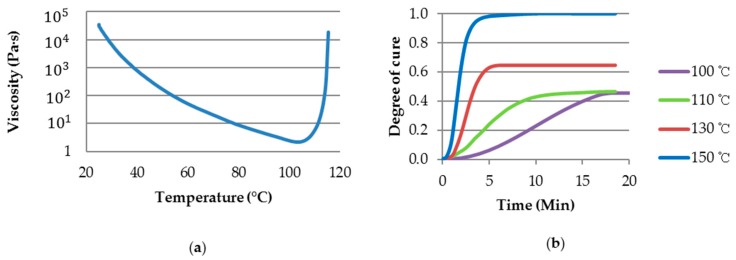
(**a**) Viscosity dynamic scan; (**b**) Degree of cure versus time at different temperatures for the fast-cure epoxy resin.

The optimum forming conditions were determined using differential scanning calorimetry (DSC, TA Q2000) and a rheometer (Anton Paar Modular Compact Rheometer 302) to identify the curing temperature time of the matrix in the prepreg. The epoxy resin began to cure after 110 °C in the temperature sweep test of the viscosity and the lowest viscosity region before the curing stage was between 95 and 105 °C, as shown Figure 3a. Therefore, 100 °C was selected as the thermoforming temperature for the epoxy/carbon prepreg because it was the temperature with the lowest viscosity. The DSC dynamic and isothermal scanning results provided the relationship between the degree of cure and time at variable temperatures (Figure 3b). The degree of cure (Equation (4)) is the ratio of the isothermal heat reaction ($H_{T}$) to the amount of heat generated during dynamic scanning ($H_{U}$). Resin is considered to be uncured when α = 0 (0%) and fully cured when α = 1 (100%) [26].

(4) $$\alpha =\frac{H_{T}}{H_{U}}$$

When the epoxy resin was exposed to a heat of 150 °C, the degree of cure was calculated as 98.5%, while was 46.6% and 64.7% at 110 °C and 130 °C, respectively. It was found that 100 °C had the lowest degree of cure (45.8%) among the 4 different temperature ranges throughout the curing process as shown in Figure 3b. When the polymer chains in of a fast-cure type epoxy resin passed at a specific point (temperature or time), a rapid cross-linking actions between the chains led to the establishment of network structures that provided the limitation on formability of prepreg.

### 3.2. Mechanical Properties of Prepreg at the Process Temperature

The tensile strength and Young’s modulus of three different prepregs were measured to demonstrate the non-linear mechanical behavior of prepreg at high temperature forming conditions. The thickness of each fiber was averaged by measuring 10 different points along the thickness direction. It was observed that UD and PW fabrics were fully impregnated by epoxy resin without any major voids, which might not affect the overall thickness of prepreg in high temperature conditions. Therefore, the assumption was made that thicknesses of three different prepregs at high temperature with the lowest viscosity were considered to be identical to the thickness of prepregs at room temperature. The thickness measurements showed that prepregs containing plain woven pattern fabric were thicker than UD carbon prepregs. Consequently, the thickness of prepreg was highly dependent on the structure of fabric.

**Figure 4:**
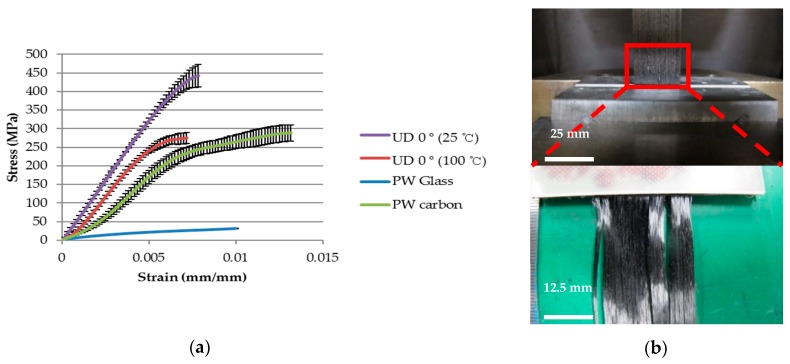
(**a**) Non-linear stress-strain curves for each prepreg at 100 °C; (**b**) Enlargement of UD 0° prepreg images after a high temperature tensile test.

Load and displacement for the prepreg was measured using a universal test machine by three times each and the stress-strain curve and the mechanical properties for each prepreg are shown in Figure 4a and Table 3. The tensile stress-strain curves for UD 0°, as illustrated in Figure 4a, show that the rigid epoxy resin within the fiber bundles at room temperature exhibits more linear behavior than high temperature of 100 °C and resultantly improves the overall tensile properties of the material. Non-linear mechanical properties of prepreg are also caused by the weaving pattern of reinforced fiber and these intrinsic behaviors of plain woven carbon and glass prepreg may be strengthened by an additional flow of resin at a high temperature. The slope of intermediate linear region in the stress-strain curve was chosen to calculate the modulus of each prepreg. Although the values of tensile strength and modulus were mentioned in Table 3, they were not able to represent the non-linear behavior of prepreg at an elevated temperature during the thermoforming simulation. Therefore, five data points averaged from the five distinguished regions of each mechanical test for prepregs were used as input data for non-linear mechanical properties in the simulation. No slippages were identified after the test and fiber fractures were observed near the grip of the specimen (Figure 4b). The mechanical properties of UD carbon 90° were measured using dynamic mechanical analysis (DMA) and its characteristic was represented by only resin properties, which were the lowest values recorded among the four prepregs. Therefore, fiber separation in the outer layer of pattern 2 containing the UD carbon is likely to occur during the forming process.

The COF for three different prepregs were evaluated with the thermoforming parameters. Table 4 shows that a suitable prepreg layer against the tool surface is included in the pattern containing the outer UD carbon layer and this layer would minimize the internal stresses of the laminate during the forming process [27]. However, a high risk of fiber separation for the UD carbon during the forming must be considered. An identical experimental procedure for the prepreg-tool surface was conducted to evaluate the prepreg-prepreg interaction with five different inter-ply combinations (Table 5). Both sides of the steel frame were used to fix the prepreg and the top and bottom surfaces of the electrically heated steel mold were covered with another type of prepreg. Three repeated measurements of friction force for five different fabric patterns were conducted. A similar trend was observed in the prepreg-prepreg patterns containing the PW carbon and glass prepregs with prepreg-tool interactions, which exhibited a higher COF for the PW carbon and glass (No. 1, 4 and 5 in Table 5) than the patterns containing only UD carbon (No. 2 and 3). Different fiber orientations between the UD carbon prepregs also increased the COF compared to the frictional force between the UD carbon and tool surface. This is because of the higher frictional force created by the different direction fibers sliding against each other. Therefore, the frictional behavior of the laminate during the forming stage is mainly dependent on the weave patterns and fiber orientations of the prepreg. Moreover, the adjacent plies containing PW carbon or glass might help preventing any fiber separations of UD prepregs that could lead to a reduction in wrinkles because of their high COF [28].

**Figure 5:**
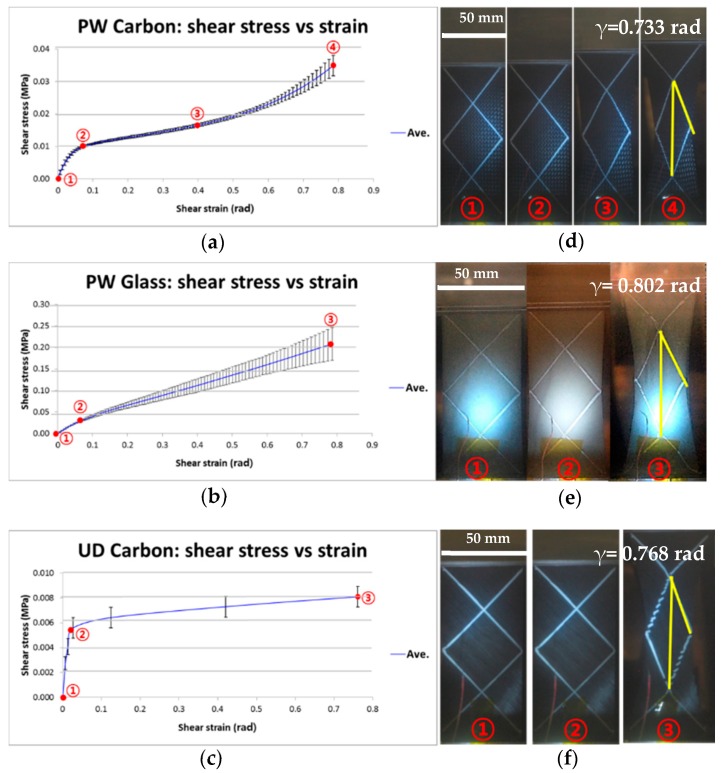
Comparison between the theoretical shear strain (angle) of for (**a**) Plain–woven (PW) carbon; (**b**) PW glass and (**c**) UD carbon prepreg and real shear angle measurements; (**d**) PW carbon; (**e**) PW glass and (**f**) UD carbon prepreg at a high temperature.

Three different prepregs were characterized using a bias extension test to study their in-plane shear behaviors under the forming conditions with a constant speed. All cross-plied specimens were deformed symmetrically and three obvious shear zones within the specimen were identified via the bias extension tests. Optical analysis of shear angle measurements obtained from the image analyzer software (Figure 5) was also conducted to compare with theoretical shear angles calculated from Equation (1). The results showed that the measured values of shear angle from three different prepregs were within the theoretical value ranges as illustrated in Figure 5. The possibility of lubricating effect in the intersections of warp and weft fiber by the resin flow would affect the shear angles obtained from the optical analysis [29].

During the load-displacement measurements, the initial force variation after three repeated measurements was low until the displacement was 6 mm and then the variation increased gradually towards the end of the experiment. The shear stress-shear strain curve for each prepreg as illustrated in Figure 5 shows that the shape depends on the weave patterns of the prepreg. The results reveal that the ideal shape of the curve in the highly shear-dominated PW carbon contains three distinctive regions including a shear locking area with two inflection points along the curve. The first inflection point is located where the shear stress is approximately 0.01 MPa and the shear strain is 0.0747 rad, which indicates a sharp increase in the stiffness during the yarn rotation 1.34 × 10^−4^ (GPa). The rotation between the warp and weft due to the shear force is limited when shear strain is 0.403 rad, which is also known as the shear locking angle [30]. The shear modulus in the shear locking angle region decreased to 2.01 × 10^−5^ GPa. Deformation beyond the locking angle leads to wrinkles in the specimen, which is caused by out-of-plane deformation or buckling. After passing second inflection points, a fractional increase in the shear modulus of 4.78 × 10^−5^ GPa was then observed upon aligning the reoriented yarns in the loading direction. The shear angle range below 0.8 rad was considered in this study, because the scattering range of the obtained shear modulus values above 0.8 rad was significantly wide to use as input data in thermoforming simulation. On the other hand, a coarse and irregular plain woven pattern of glass (PW glass) causes more indefinite inflection points and shear locking areas in the shear stress-shear strain curve in comparison with PW carbon. In the case of the UD carbon, the plateau region of the graph after a sharp increase in the stiffness indicates that the fabric straightens without interference between the bundles [10].

### 3.3. Square-Cup Drawing Test

The formability of the prepreg laminate was analyzed via drawing tests using the specified design of the base mold, punch and holder. Before the main forming operation, the input for the main temperature controller was set at 100 °C for the punch and chamber, 90 °C for the holder and 85 °C for the base mold to ensure the set temperature was not exceeded.

**Figure 6:**
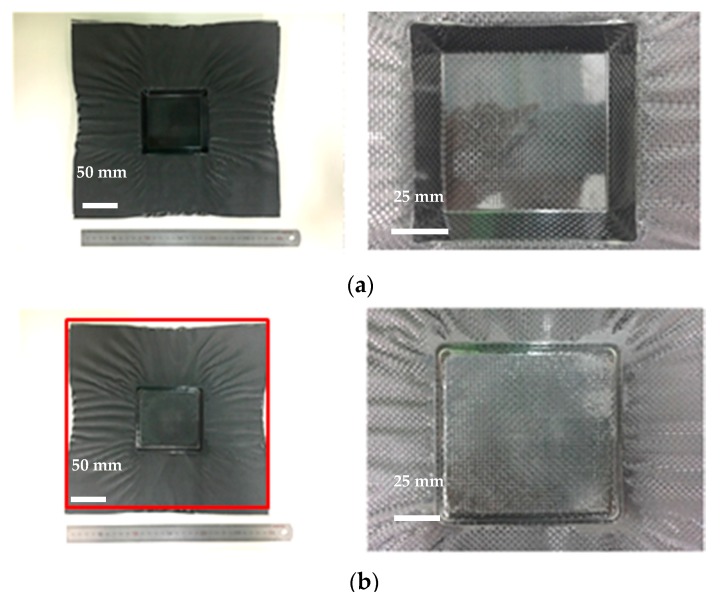
(**a**) Inner layer and (**b**) outer layer of the thermoformed square-cup for the Type 1 mold design with the pattern 1 prepreg laminate.

**Figure 7:**
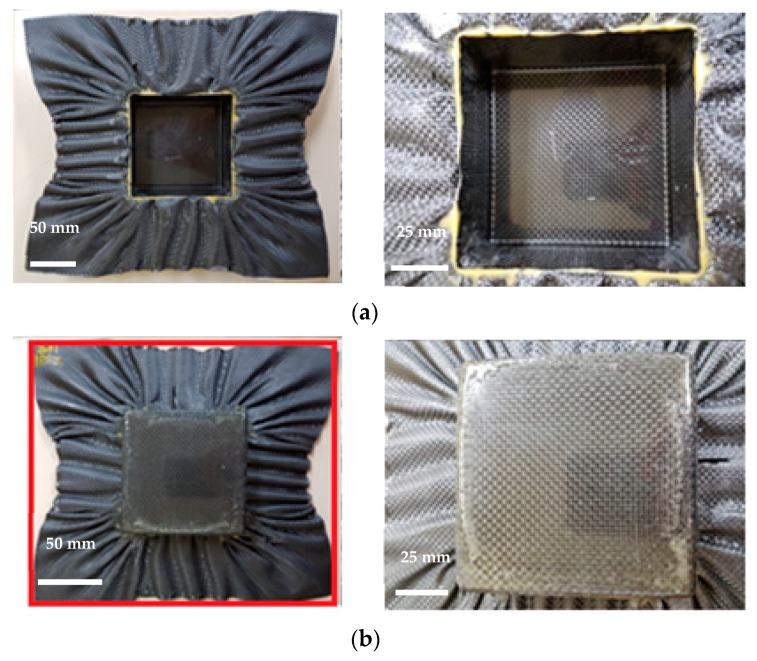
(**a**) Inner layer and (**b**) outer layer of the thermoformed square-cup for the Type 2 mold design with the pattern 1 prepreg laminate.

**Figure 8:**
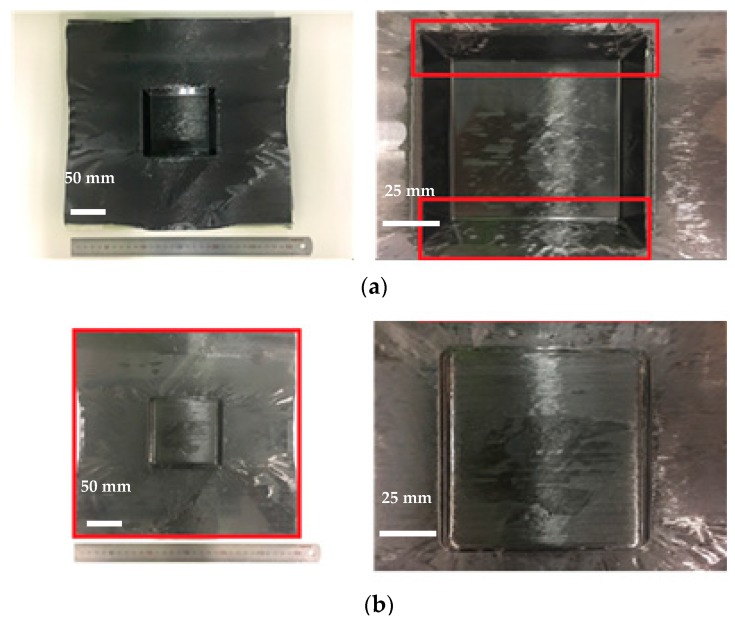
(**a**) Inner layer and (**b**) outer layer of the thermoformed square-cup for the Type 1 mold design with the pattern 2 prepreg laminate.

The views from both sides of the thermoformed square-cup with the pattern 1 prepreg laminate using the Type 1 mold are shown in Figure 6. The dimensions of the prepreg laminate in Figure 6b also show that the middle sections of each side line were drawn into the center by a few millimeters. This contraction made it possible to demold the formed square-cup without any significant external stresses. Flat and smooth surfaces were obtained on the inclined wall of the square-cup after demolding. However, excessively cured epoxy layers at the corners and edges were present on the outer and inner layer, respectively. The large resin content on the surface of the laminate (PW carbon) and the high flow of the resin-rich areas inside the prepreg laminate during the thermoforming caused the highly pressed epoxy resin to agglomerate at the corner of the square-cup shaped part. On the other hand, an identical prepreg pattern laminate with the Type 2 mold design shown in Figure 7 involved a deeper drawing action. More wrinkles and shrinkages around the square-cup were observed. The process was found to be more time-consuming than type 1 mold for demolding the final formed product. It was thought that shrinkage of formed product was likely to occur around the horizontal centerline of product. This would create an increased amount of contact pressure and friction between the product and mold if a proper tilting angle was not provided. Therefore, determination of the ideal tilting angle for a given mold was very important factor to improve the demolding process. The square-cup with the Type 2 mold had more excessive epoxy resin at the edges of the inner and outer layers than the Type 1 mold. Therefore, to meet the Class A surface quality for automotive products, a non-uniform resin distribution on the surface of the formed product due to the resin-rich area must be taken into consideration when the compositions of the prepregs and design of molds are determined. Although there were some difficulties to identify the shear deformation in the formed pattern 1 against Type 2 mold, Wang et al. [31] studied the yarn reorientation of plain woven carbon fabric with square-shaped punch during the preforming process. The study showed that shear angles at the corner of square box were between 50 ° and 60 °. Therefore, high shear angles in pattern 1 with Type 2 mold would had been affected by a deeper drawing action. When pattern 2, which contained UD carbon prepreg on the outer layer, was formed against a Type 1 mold, fiber separations and distortions (wrinkles) occurred on both sides of the square-cup (Figure 8). This is due to the orientation of the UD carbon at 90 ° with respect to the punching direction (downwards). Only shrinkage of the fiber orientation at 0° (left and right side of the laminate in Figure 8b) appeared after thermoforming. The amount of the excessively cured resin layers was reduced at the corners and edges of the square-cup. Therefore, the amount of resin on the outer layer of the pattern 2 laminate can be regarded as the optimum amount of resin in the thermoforming process to prevent epoxy resin being agglomerated. However, from an aesthetic point of view, pattern 2 is not the optimum composition to thermoform the square-cup.

**Figure 9:**
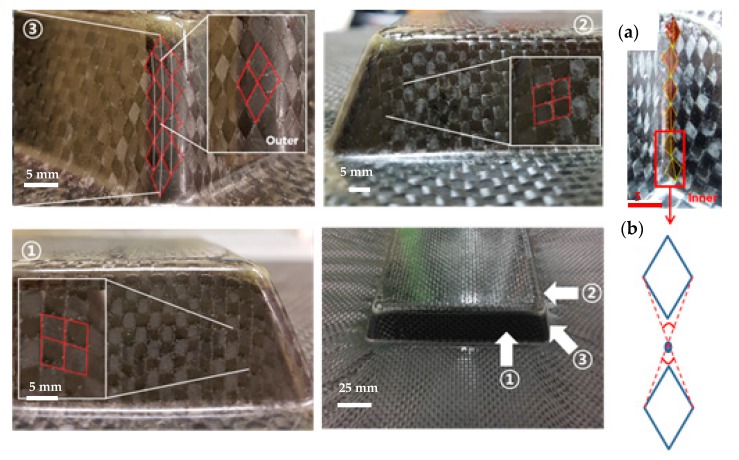
Shear-dominated regions of the PW carbon prepreg at ①, ② and ③, (**a**) Shear angle measurement at the inner corner of ③, (**b**) Averaging the shear angle by connecting imaginary lines between two rhombic shapes.

The evidence at the four corners of the square-cup suggested that the formability of the outer layer of the PW carbon prepreg in the pattern 1 laminate was highly shear-dominated during thermoforming. Three different regions at the corner of the outer PW carbon layer are illustrated in Figure 9 and the deformations of the yarns in the PW carbon gradually shifted towards the center line of the corner where the most severe deformation occurred via shear action (Region ③ in Figure 9). However, the variations in the upper and lower angles of the deformed fabric yarns in the inner and outer layer were too wide to use as conclusive results. This might be caused by the asymmetric shear deformations of the fabric during the thermoforming process. It was thought that the scattered data also depended on the uniformity of the weaving strength and number of yarns per bundle in the PW carbon. The average values of the upper and lower angle in each rhombic shape were used to calculate the shear angles. Some of the yarns in the inner layer slipped out of place and the discontinuity of the rhombic patterns was identified in the region where the most extreme shear was experienced (Figure 9a). To estimate the angle in this case, the mid-point between two rhombic shapes was designated by connecting imaginary lines in a diagonal direction to measure the angle between the warp and weft yarns (Figure 9b). A general trend of the experimental results shows that the shear angle increases for the first 10 mm of the path and reaches the highest value between 10 mm and 15 mm on the path. The shear angles between the outer and inner layers of the PW carbon did not coincide each other at each corner of the square-cup.

**Figure 10:**
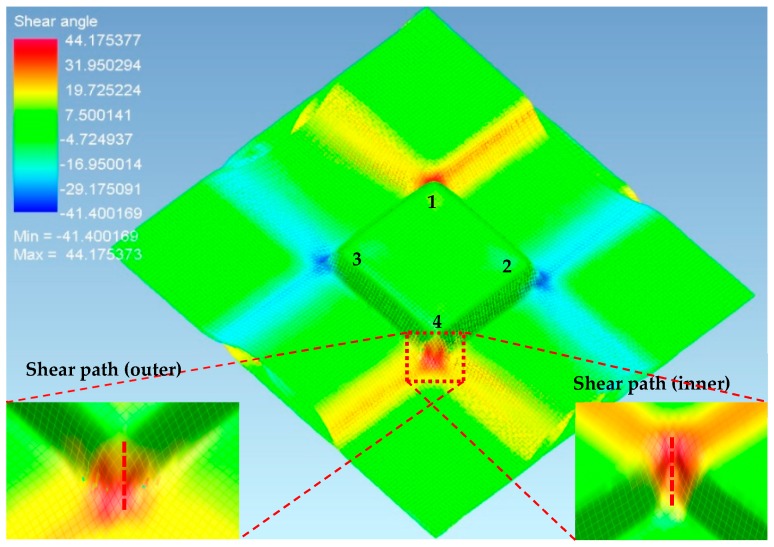
Predicted shear angles in the Type 1 mold design with the pattern 1 prepreg laminate at corner 1, corner 2, corner 3, corner 4 using the PAM-FORM simulation (From top view).

**Figure 11:**
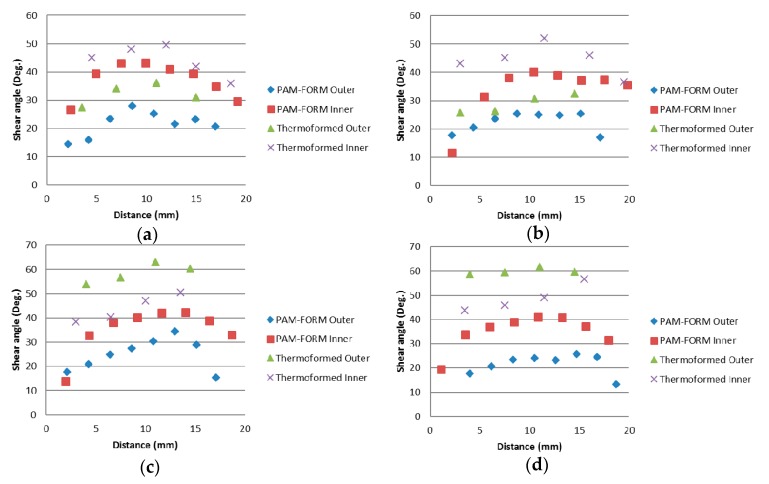
Comparison between the experimental and simulation results for the shear angle at (**a**) corner 1; (**b**) corner 2; (**c**) corner 3; (**d**) corner 4 as illustrated in Figure 10.

The virtual forming simulation for thermoforming of the square-cup was implemented in PAM-FORM to compare the deformation behavior of the prepreg laminate with the experimental data. The most severe shear deformations experienced during the forming simulation were represented by the red and blue zones as illustrated in Figure 10 and the predicted shear angle in each element was considered as an absolute value. Each element (rhombic shape) was then analyzed to find the major diagonal distance (path length) and shear angle. The refinement level of mesh at the center of material layers where meets punch was higher than outside of the square cup in order to achieve high accuracy of shear deformations at the corners of square cup. This could be the one of the reason why wrinkles were not predicted in the outside region, as illustrated in Figure 10. An attempt to simulate the Type 2 mold was unsuccessful due to the high number of wrinkles around the square-cup from the deeper drawing action at the center of the laminate, which was seen in the experimental results. The simulation data had a tendency to increase the shear angle along the corner of the square-cup with a less scattered data than the experimental ones, which can be seen in Figure 11. Superimposing all the experimental and simulation data in a single graph shows the quantitative comparison of the shear angle along a path at the corners. The average shear angle in the experimental results was higher than that in the simulation results and overlapping data between 10 mm and 20 mm were identified at corners 1 and 2 on both sides of the laminate. The gradual increase in the shear angle for the first 20 mm path of the simulation in Figure 11 described the experimental behaviors well. Differences between the forming simulation and experimental results might be caused by a relatively large mesh sizes used in material properties for PAM-FORM simulation to decrease the computation time. The errors were also occurred during the averaging scheme of shear angle used to determine the value for irregular deformation patterns.

## 4. Conclusions

The effects of the structural parameters on the manufacturability of fast-cured epoxy resin/carbon prepregs were investigated to optimize the thermoforming process for a mass production line in the automotive industry. The thermoforming process conditions were determined by analyzing the properties of the epoxy resin using differential scanning calorimetry (DSC) and a rheometer. The lowest epoxy viscosity was maintained at 100 °C for 20 min. Therefore, we decided that the best temperature condition at the thermoforming stage was 100 °C, which was then applied to high-temperature mechanical tests. The development of test methods to measure the non-linear mechanical behaviors, such as tensile, in-plane shear (intraply) and frictional force, within the prepregs (interply) for UD carbon, PW carbon and PW glass were introduced. It was expected that a more realistic and predictive virtual forming simulation would be obtained using the input data from non-linear mechanical tests. Two different square-cup shaped molds, type 1 with a tilting angle of 110° and depth of 20 mm and type 2 with a tilting angle of 90° and depth of 40 mm, were prepared to reproduce the shape of a vehicle’s roof panel on a small scale. The final product of the thermoformed square-cup proved that the formability and demoldability of the prepreg are affected by the geometry of the mold. Two different stacking sequences of the prepreg laminates were used to assess the effect of formability on the extreme curvature parts, which are the most vulnerable areas to form. Experimental and PAM-FORM simulation data for the shear deformations at each corner were compared to confirm the similarity of the overall tendency of the shear angle in the first 20 mm of the path at each corner.

## Figures and Tables

**Table 1:** Physical properties of the different types of prepreg.

Model Name	Type	Thickness (Standard Deviation) (mm)	Resin Content (vol %)	Weight (g/m^2^)
7628	Plain woven (PW) glass	0.305 (±3.1 × 10^−6^)	42	209
CF-3327	Plain woven (PW) carbon	0.269 (±5.7 × 10^−6^)	42	200
CU-190	Unidirectional (UD) carbon	0.202 (±3.8 × 10^−6^)	38	190

**Table 2:** Description of the stacking sequences for thermoforming tests.

Ply	Pattern 1	Pattern 2
1	Carbon PW	UD 0°
2	UD 0°	UD +45°
3	UD +45°	UD −45°
4	UD −45°	Glass PW
5	Glass PW	Glass PW
6	Glass PW	Glass PW
7	UD −45°	Glass PW
8	UD +45°	UD −45°
9	UD 0°	UD +45°
10	Carbon PW	UD 0°

**Table 3:** Average and standard deviation of strength and Young’s modulus from three repeated measurements for unidirectional (UD) 0° (red), PW carbon (green), PW glass (blue) and UD carbon 90° at 100 °C.

Prepreg	UD Carbon 0°	PW Carbon	PW Glass	UD Carbon 90°
Strength (MPa)	280.1 (±18.3)	72.2 (±5.3)	30.0 (±1.1)	1.4 × 10^−3^ (±1.6 × 10^−4^)
Modulus (GPa)	60.4 (±3.7)	11.2 (±1.1)	0.57 (±0.04)	2.9 × 10^−2^ (±2.6 × 10^−3^)

**Table 4:** Coefficient of friction between tool and prepreg with three repeated friction force measurements at the thermoforming conditions.

Prepreg	UD Carbon 0°	UD Carbon 90°	PW Carbon
Coefficient of friction	0.050	0.063	0.202
Standard deviation	0.007	0.010	0.011

**Table 5:** Coefficient of friction of five different prepreg-prepreg patterns against our process parameters.

No.	Interface	Coefficient of Friction (COF)	Standard Deviation
1	PW carbon/UD 0°	0.180	0.002
2	UD 0°/UD +45°	0.078	0.002
3	UD +45°/UD −45°	0.074	0.005
4	PW glass/UD −45°	0.162	0.004
5	PW glass/PW glass	0.157	0.004
